# The soft X-ray spectromicroscopy beamline BL08U1A upgrade at SSRF

**DOI:** 10.1107/S1600577524006684

**Published:** 2024-08-22

**Authors:** Xiangjun Zhen, Zhi Guo, Zengyan Zhang, Yong Wang, Renzhong Tai

**Affiliations:** a Shanghai Synchrotron Radiation Facility, Shanghai Advanced Research Institute Chinese Academy of Sciences Zhangheng Road 239 Pudong District Shanghai201204 People’s Republic of China; ESRF – The European Synchrotron, France

**Keywords:** elliptical polarization undulator, photon flux, energy resolution, variable-line-space plane grating

## Abstract

Beamline BL08U1A is a soft X-ray spectromicroscopy beamline at Shanghai Synchrotron Radiation Facility (SSRF) that exhibits the capabilities of high spatial resolution (30 nm) and high energy resolving power (over 10^4^). As a first-generation beamline of SSRF, owing to its continuous operation over the last ten years, an urgent upgrade of the equipment including the monochromator was deemed necessary. The upgrade work included the overall construction of the monochromator and replacement of the mirrors upstream and downstream of the monochromator. Based on its original skeleton, two elliptically cylinder mirrors were designed to focus the beam horizontally, which can increase the flux density by about three times on the exit slits. Meanwhile, the application of variable-line-space gratings in the monochromator demonstrates the dual functions of dispersing and focusing on the exit slits which can decrease abberations dramatically. After the upgrade of the main components of the beamline, the energy range is 180–2000 eV, the energy resolving power reaches 16333 @ 244 eV and 12730 @ 401 eV, and the photon flux measured in the experimental station is over 2.45 × 10^9^ photons s^−1^ (*E*/Δ*E* = 6440 @ 244 eV).

## Abstract 2

The upgrade of beamline BL08U1A at SSRF has been recently conducted. The energy range is 180–2050 eV, the energy resolution can reach up to 16333 @ 244 eV and 12730 @ 401 eV, and the photon flux measured in the experimental station is over 2.45 × 10^9^ photons s^−1^ (*E*/Δ*E* = 6440 @ 244 eV).

## 1. Introduction

The BL08U1A beamline at Shanghai Synchrotron Radiation Facility (SSRF) has been in operation since 2009. This beamline contains an elliptical polarization undulator (EPU), front-end, entrance slit, cylindrical front-focusing mirror, monochromator, toroidal rear-focusing mirror, exit slits, *etc*. The main function of a scanning transmission soft X-ray microscopy (STXM) system is to obtain the chemical state distribution of samples (Stöhr, 1992 ▸). A monochromatic beam is focused onto the sample by a zone plate with a spot size of less than 30 nm, where the +1st order is sorted by an order-sorting aperture. The STXM system includes zone plate, sample stages, detectors, *etc*. The EPU provides a higher photon flux and higher coherent X-rays than a bending-magnet source. The EPU radiation in the fundamental and the third harmonic is available for the requirements of lower (200–800 eV) and higher (800–2000 eV) energies. A pink-beam entrance slit limits the incident angle and absorbs partial radiant heatload. A sagitally cylindrical mirror (M1) collimates the incident light parrallelly in the vertical direction. A traditional SX700 type is selected for the monochromator (Reininger & Castro, 2005 ▸) to cover a relatively broad photon energy range, where the optical design is based on a variable-including-angle plane-grating monochromator. It contains two equal-spacing gratings (800 lines mm^−1^ and 1200 lines mm^−1^) on the same substrate and an internal cooling plane mirror to provide monochromatic light with high energy resolution (Gong *et al.*, 2013 ▸). A toroidal mirror (M3) focuses monochromatic beam to the exit slits in the meridional and sagittal directions. The exit slits can be considered as a secondary light source, and monochromatic light with specific energy resolution is taken from the size of the opening. The secondary light source of the exit slits is focused onto the sample by the zone plate which can provide monochromatic light with spatial resolution better than 30 nm.

Beamline operation time is about 7000 h each year, and long-term overload operation can lead to an acceleration of the wear and tear of key equipment, *e.g.* the monochromator. Carbon contamination of the optics, wearing of the mechanical structure, degradation of the rotational accuracy and repeatability, for example, all cause a decrease in the energy resolution. And because two gratings are engraved on one substrate, there are few choices of manufacturers who can provide the same equal-spacing type of gratings. Therefore, it is more difficult and costly to update and maintain the monochromator. Aberration caused by the cylindrical-shape sagittal focusing mirror is another cause of the degradation of the energy resolution and photon flux on the sample. Therefore, in order to upgrade the beamline performance to fulfill the requirements for STXM experiments, beamline redesign is inevitable, including the replacement of the monochromator, pre-focusing and post-focusing mirrors, *etc*.

The beamline upgrade has been accomplished: the new monochromator has been designed to rely on a variable-including-angle mechanism with one inner cooling plane mirror and two variable-line-space gratings; the sagittal pre-focusing mirror (M1) with cylindrical shape and rear-focusing mirror (M3) with toroidal shape have been replaced by two meridional focusing mirrors with elliptical and cylindrical shape. The EPU light source and STXM endstation are not included in this upgrade. After the upgrade, the photon energy of the beamline has been broadened to 180–2000 eV, the photon flux in the sample position has been increased to 2.45 × 10^9^ photons s^−1^ (*E*/Δ*E* = 6440 @ 244 eV), and the energy resolving power has been increased to 16333 @ 244 eV and 12730 @ 401 eV.

## 2. Beamline

### 2.1. EPU source

**Figure 1:**
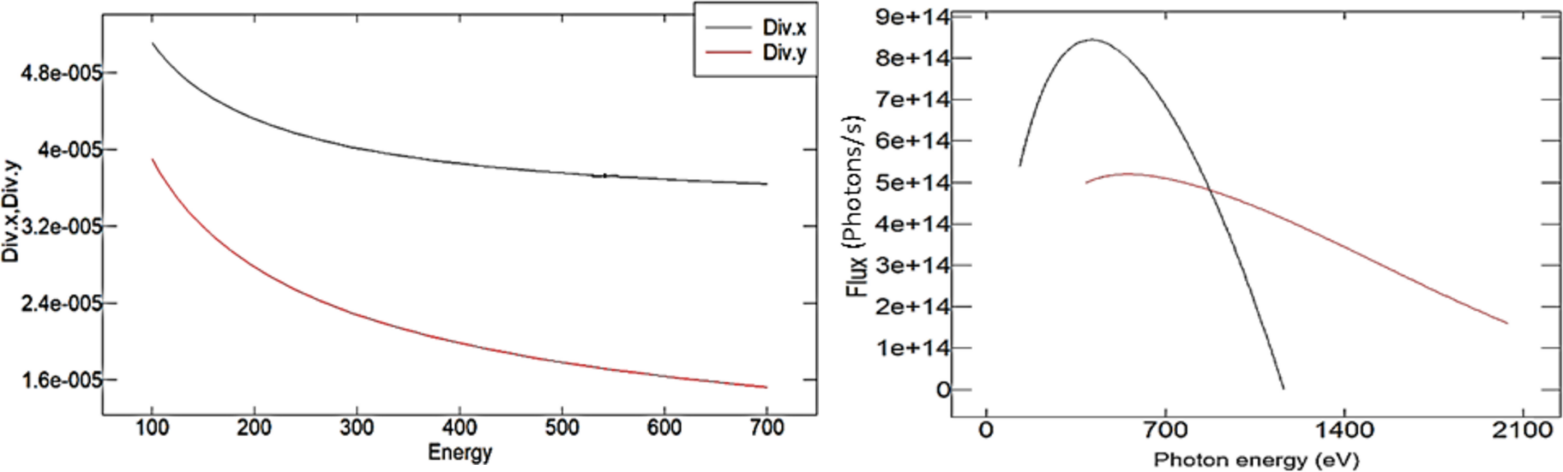
Divergence (left) and photon flux (right) of the EPU light source.

SSRF is a third-generation synchrotron with an electron energy of 3.5 GeV. The light source of beamline BL08U is located in a standard linear section. Considering practical issues such as the manufacturing process and thermal load handling, an EPU was chosen as the light source for STXM, with period length 10 cm and number of periods 42. The source divergences for ∑*x* and ∑*y* are 45.7 µrad and 31.6 µrad, respectively; the spot size is 159 µm (h) × 24 µm (v) @ 150 eV. The first harmonic of EPU radiation can be selected for the energy range 100–700 eV, and the third harmonic for 700–2000 eV. The maximum horizontal and vertical divergence angles are 0.22 mrad (4.7∑*x*) and 0.15 mrad (4.7∑*y*), respectively. Fig. 1 ▸ shows the horizontal and vertical divergence and photon flux of the light source.

### 2.2. Beamline upgrade design

The original optics has an acceptance angle of 0.16 mrad (h) × 0.08 mrad (v). The pre-focusing mirror (M1), monochromator and rear-focusing mirror (M3) are located 30 m, 32 m and 34 m downstream of the source point, respectively. The beamline is collimated in the vertical direction by M1 and focused in the horizontal and vertical directions by M3. Their meridional slope errors are 2–3 µrad, leading to a large aberration in the exit slits and focusing point which affects the focusing spot uniformity, photon flux and energy resolution *etc*. The monochromator contains an internally cooled plane mirror and two gratings (800 lines mm^−1^ and 1200 lines mm^−1^).

**Figure 2:**
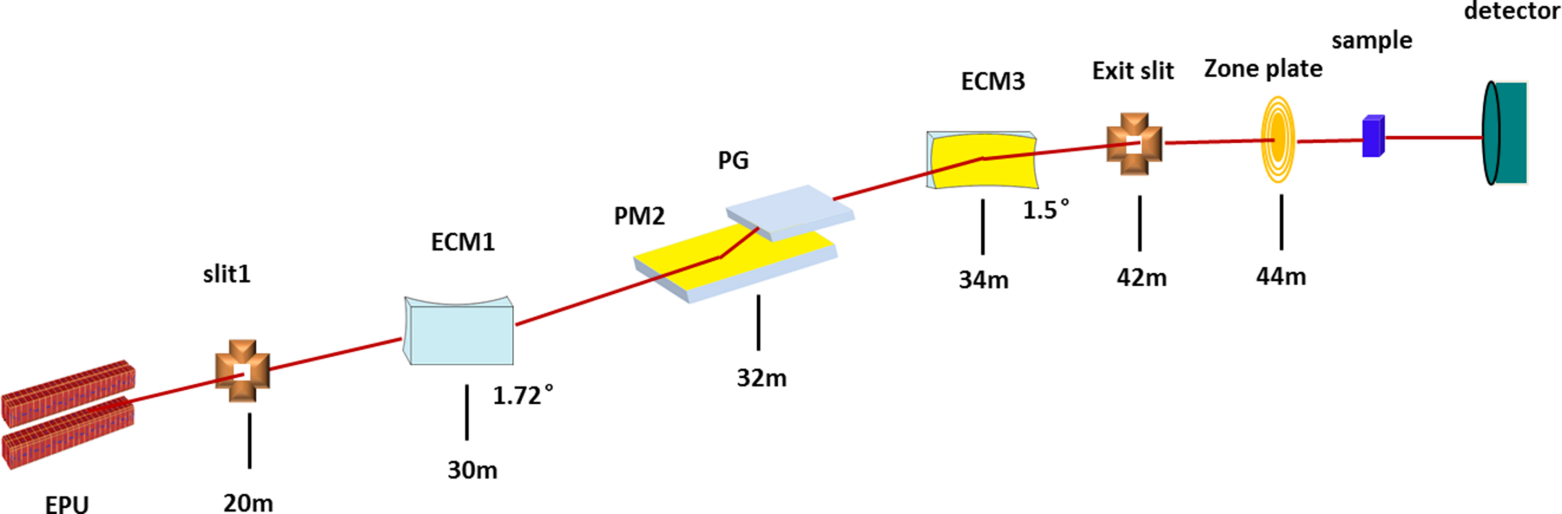
Layout of the new BL08U1A beamline at SSRF. The original front cylindrical mirror and toroidal mirror are replaced by meridional focusing elliptical cylindrical mirrors, and the monochromator has been replaced by a variable-included-angle varied-line-spacing plane-grating monochromator.

**Figure 3:**
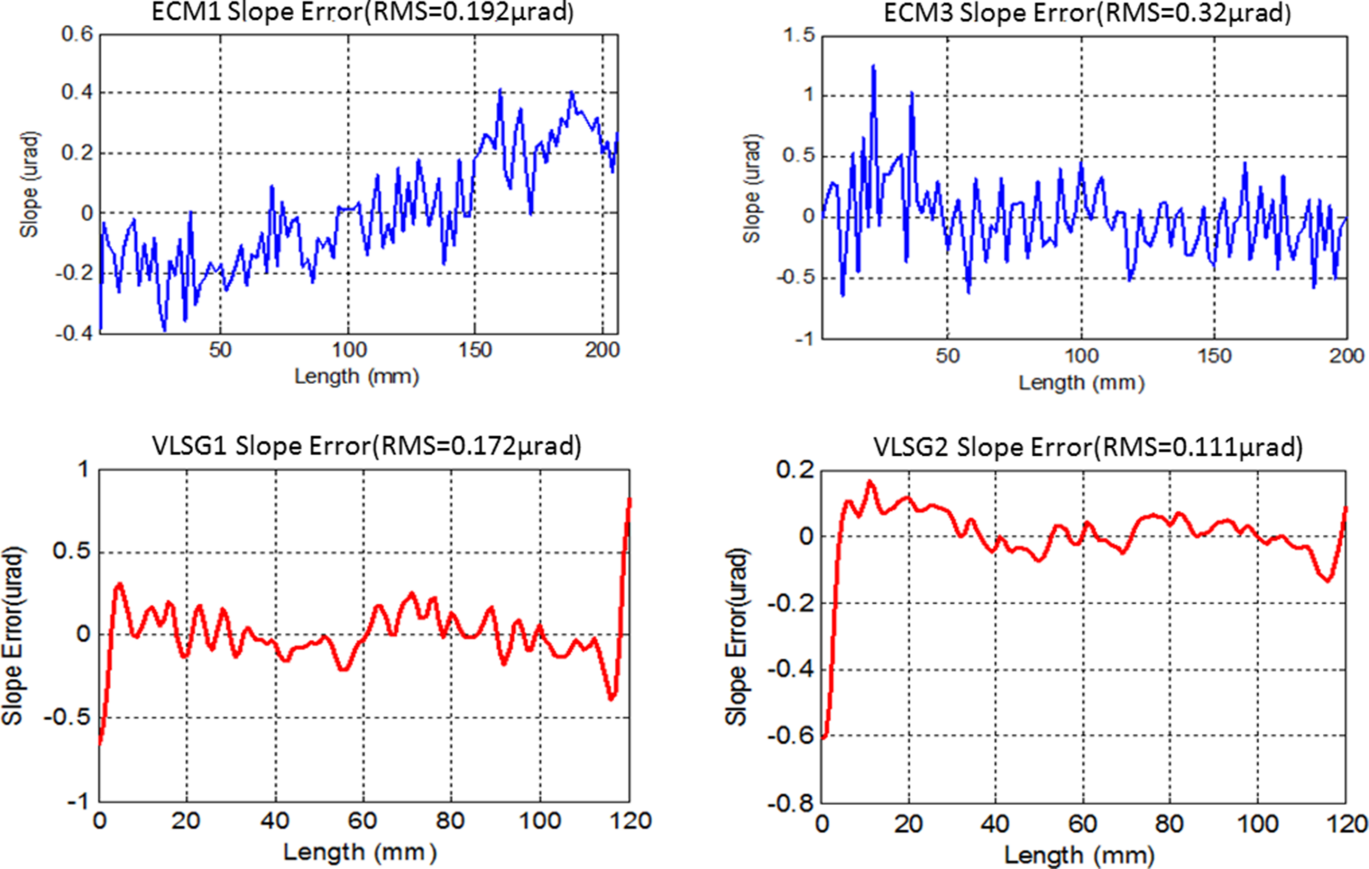
Slope error measurement results using an autocollimator-based long trace profiler.

Fig. 2 ▸ shows the updated optical layout. The basic layout of the beamline (*i.e.* positions of the optical components and incidence angle, *etc*.) remains unchanged. The original collimating mirror in the sagittal direction has been replaced by an elliptically focusing mirror (ECM1) in the meridional direction, located 30 m downstream of the light source with a grazing incidence angle of 1.72°. The beam is focused 33 m downstream of the source in the horizontal direction. The original plane mirror PM2 of the monochromator is still utilized, while the original grating (32 m downstream of the light source) has been replaced by a variable-line-spacing grating. The dispersed diffraction beam in inside order (32 m from the light source) is selected with both dispersion and focusing functions. The dispersed beam is focused onto the exit slits (42 m from the light source) in the vertical direction. Two variable-line-space plane gratings (400 lines mm^−1^ and 1000 lines mm^−1^) are mounted in the new monochromator. The original toroidal mirror (34 m from the light source point) has been replaced by a meridional elliptical cylindrical mirror ECM3, focusing the beam horizontally onto the exit slits. Compared with the original single focusing of the toroidal mirror, the dual focusing of two elliptical cylinder mirrors with slope error of less than 0.5 µrad in the meridional direction has reduced the aberration effects dramatically. The beam divergence angle in the horizontal direction downstream of the exit slits has been reduced from 1.9 mrad to 0.5 mrad, which can increase the photon flux by more than three times. The mirror parameters are detailed in Table 1 ▸. Slope error test results for the mirrors and gratings are shown in Fig. 3 ▸. In summary, the new beamline design can improve the photon flux and signal-to-noise ratio on the sample dramatically.

### 2.3. Beamline endstation

**Figure 4:**
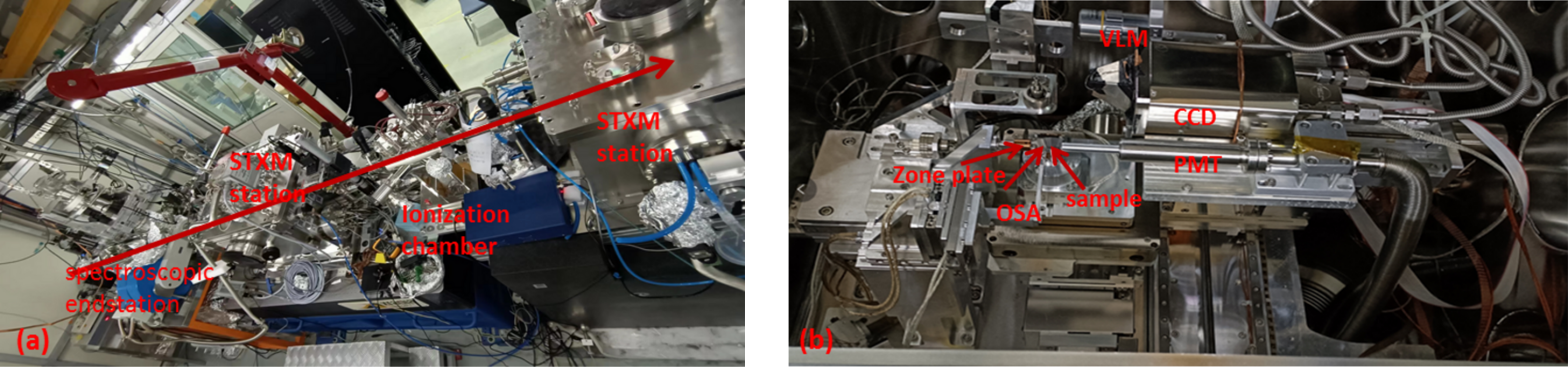
(*a*) Layout of the BL08U1A station; (*b*) STXM endstation view.

The photon energy of this beamline covers 180–2000 eV. The majority of the equipment in the experimental endstations, such as the focusing, sample and detector systems, are mostly placed in a vacuum chamber. Fig. 4 ▸(*a*) shows three endstations which consist of the STXM and the spectroscopic endstations. The spectroscopic endstation chamber is mounted at the end of the beamline with a vacuum level of ∼1 × 10^−9^ Torr. This endstation only performs spectroscopic experiments of total electron yield (TEY) with a variety of *in situ* conditions, such as providing the voltage, magnetic field and temperature control conditions (Liu *et al.*, 2019 ▸). The vacuum value of the STXM endstation is 1.33 × 10^−3^ Pa or helium environment. The vacuum chamber is separated from the beamline vacuum tube by a Si_3_N_4_ window of thickness 100 nm and size 500 µm × 500 µm (Zhang *et al.*, 2015 ▸). The STXM endstation mainly consists of the zone plate focusing system, sample scanning system, phosphor-scintillated photomultiplier (PMT) detector, sample holder, control system and graphical user interface. The spatial resolution of the STXM can reach up to 20 nm, and various experimental methods can be used, such as stack scan, coherent diffraction imaging, *etc*. (Zhang *et al.*, 2010 ▸).

## 3. Beamline upgrade design spection

### 3.1. Energy resolution

The main components that affect the energy resolution (Gong *et al.*, 2016 ▸; West & Padmore, 1987 ▸; Strocov *et al.*, 2010 ▸) include the source size, divergence of the exit slits, slope errors of the grating and plane mirror, and optical aberration, which can be represented by the following formulas, respectively,
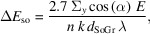

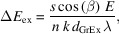

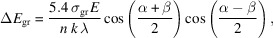

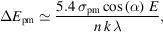

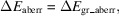

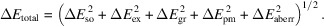
Here Σ_*y*_, *E* and λ represent the RMS values of the vertical source size, the photon energy and wavelength, respectively, α and β represent the incidence and diffraction angles relative to the normal line of the grating, *n* and *k* represent the diffraction order and the grating line density, respectively. *d*_SoGr_ and *d*_GrEx_ represent the source to grating and grating to exit slits distances, respectively, and σ_gr_ and σ_pm_ represent the slope errors of the grating and plane mirror, respectively.

**Figure 5:**
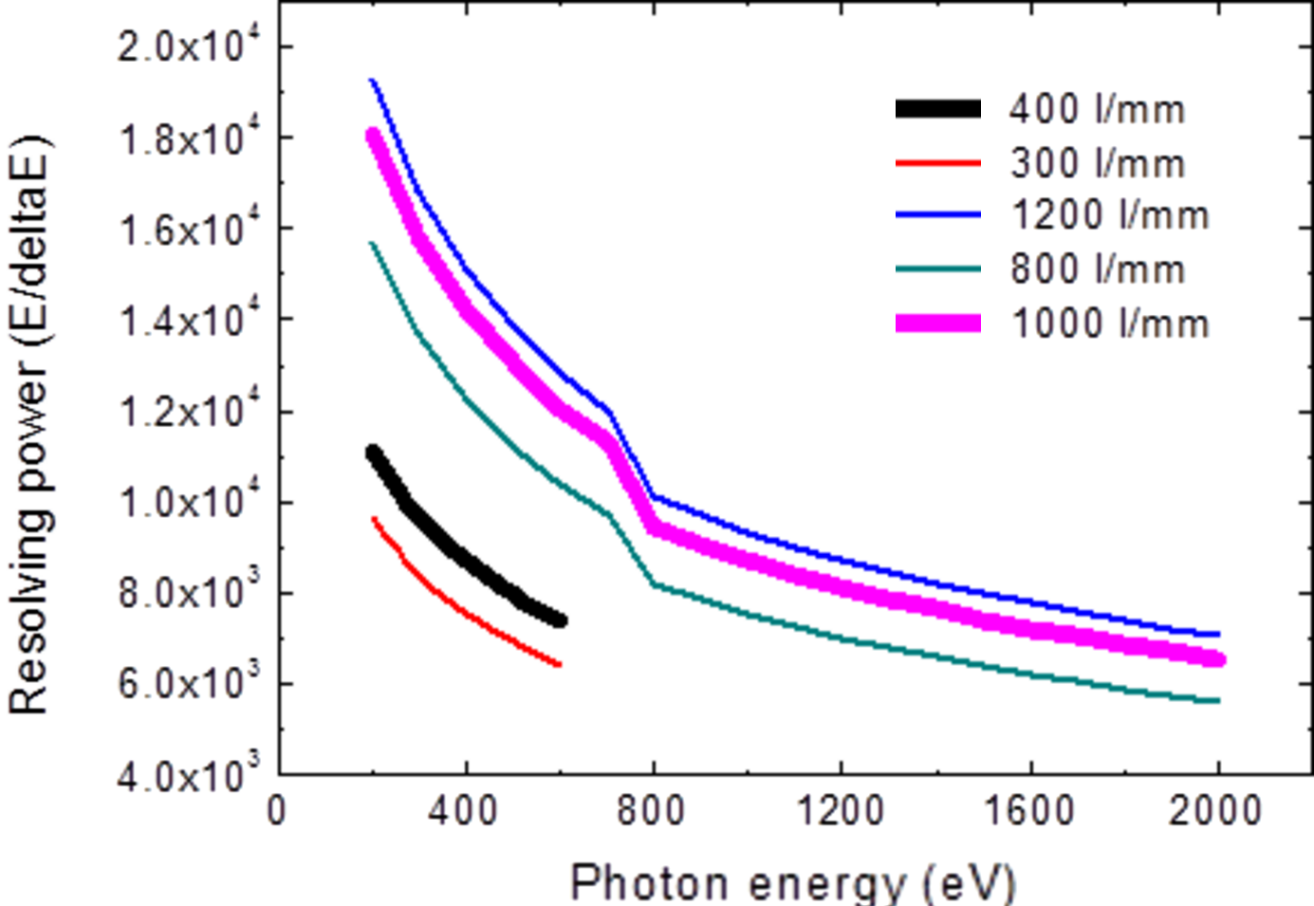
Energy resolving power for different gratings.

Under the condition that the exit slits width is 20 µm and the tangential slope error of the grating and plane mirror is 0.2 µrad, the energy resolutions for different grating line densities are shown in Fig. 5 ▸. The resolving power shows an abrupt decrease between 700 and 800 eV due to selection from the first harmonic to third harmonic of the EPU radiation, where the source size for the third harmonic radiation is relatively larger compared with that of the first harmonic.

**Figure 6:**
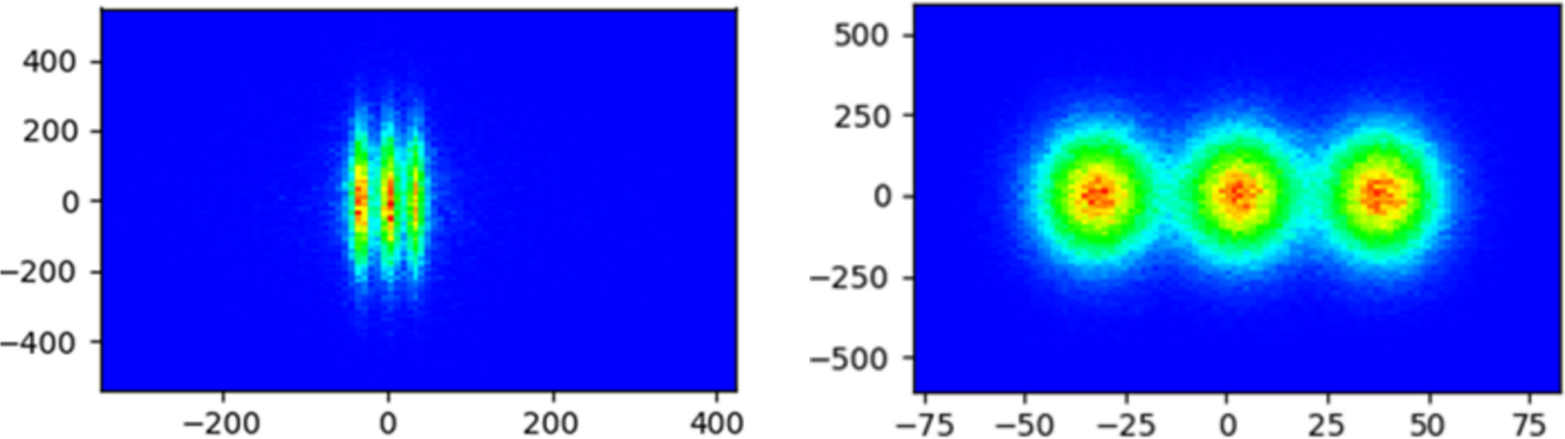
Tracing of the energy resolving power. The energy resolving power can reach up to 1.6 × 10^4^ @ 400 eV with the 400 lines mm^−1^ grating (left) and 10^4^ @ 900 eV with the 1000 lines mm^−1^ grating (right).

Based on the requirements of the experiments, two variable-line-space gratings were chosen – 400 lines mm^−1^ and 1000 lines mm^−1^. Dual focusing mode by two elliptical cylinder mirrors in the meridional direction is considered. The energy resolving power traced by *OASYS* (Rebuffi *et al.*, 2020 ▸) is shown in Fig. 6 ▸. The energy resolving power can reach up to 1.6 × 10^4^ @ 400 eV with the 400 lines mm^−1^ grating and 10^4^ @ 900 eV with the 1000 lines mm^−1^ grating.

### 3.2. Photon flux

**Figure 7:**
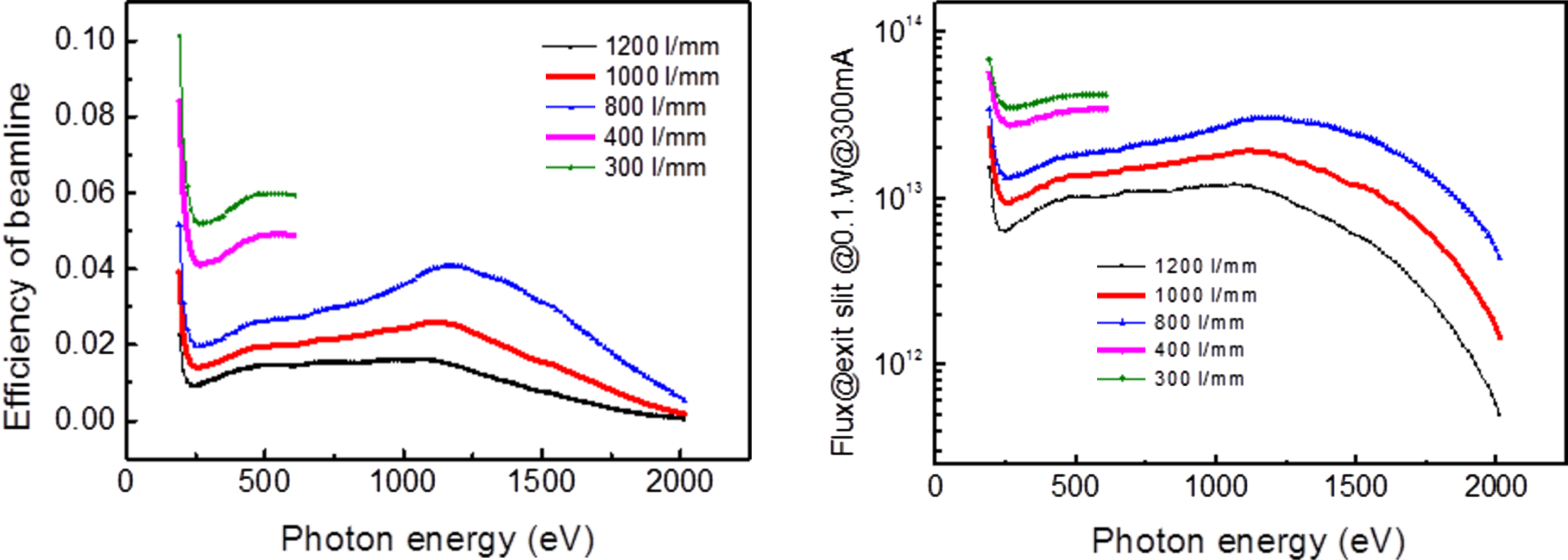
Transmission efficiency (left) and photon flux at the slits (right) of the beamline.

The transmission efficiency and photon flux of the beamline are mainly determined by the transmission efficiencies of the mirrors and gratings. The grazing incidence angles of ECM1 and ECM3 are 1.72° and 1.5°, respectively. Both mirrors are coated with gold of thickness 40 nm and roughness (RMS) 0.3 nm. The geometry of the two gratings is lamellar. The variable-line-density formula (Reininger, 2011 ▸; Meng *et al.*, 2019*a* ▸; Guo *et al.*, 2017 ▸) for the two gratings is
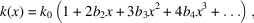
where *k*_0_ is the line density at the grating center, *x* is the coordinate along the grating length and *b*_2_, *b*_3_ and *b*_4_ are the varied-line-spacing parameters. The groove depth of the grating affects the transmission efficiency of the grating dramatically. In order to optimize the transmission efficiency, the gradient groove depth was chosen for the variable-line-space grating. The inside diffraction order with a *C*_ff_ value of 0.6 was chosen to broaden the beam-covering area on the grating and increase the transmission efficiency. Considering the reflectivity of the mirrors (ECM1 and ECM3) and the efficiency of the gratings, the beamline transmission efficiency under different line density gratings is shown in Fig. 7 ▸ (left). The new monochromator has three grating holders, while two gratings are installed. The depth range of the 400 lines mm^−1^ grating groove is 13–20 nm, and the transmission efficiency can reach up to 4%; the depth range of the 1000 lines mm^−1^ grating groove is 5–15 nm, with the beamline transmission exceeding 1% for the higher photon energy.

Based on the transmission efficiency and photon flux of the EPU source, the photon flux downstream of the exit slits is shown in Fig. 7 ▸ (right). For the 400 lines mm^−1^ grating, the photon flux at exit slits can reach up to 3 × 10^13^ photons s^−1^ (0.1% bandwidth)^−1^, and, for the 1000 lines mm^−1^ grating, the photon flux can reach up to 10^13^ photons s^−1^ (0.1% bandwidth)^−1^.

## 4. Experimental results

### 4.1. Energy resolution

**Figure 8:**
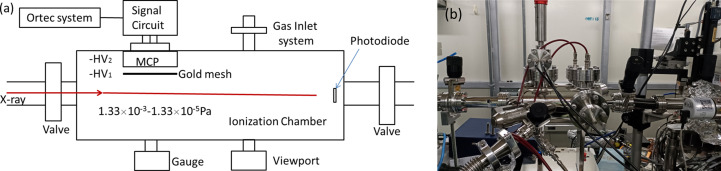
Sketch (*a*) and photograph (*b*) of the ionization chamber based on an MCP.

An ionization chamber has been designed for testing the energy resolving power, and installed downstream of the STXM endstation. The ionization chamber mainly depends on a microchannel plate (MCP) to record the total ion yield spectra (Li *et al.*, 2016 ▸). In addition, a vacuum system, Au mesh and MCP high-voltage power supply system, gas inlet system and ORTEC data acquisition for the MCP have been designed. A sketch and photograph of the chamber are shown in Fig. 8 ▸. The ionization chamber can be filled with argon or nitro­gen gas to obtain corresponding excitation spectra. When the gas pressure in the ionization chamber is stable at 6 × 10^−7^ Torr and the MCP high voltage is 2400 V, excitation spectra with good signal-to-noise ratio are collected. The exit slits size is 50 µm × 20 µm when the ionization chamber is filled with argon, and 50 µm × 10 µm when filled with nitro­gen. The excitation spectrum is fitted by a Voigt profile to obtain the corresponding Gaussian width and to obtain the corresponding energy resolving power.

**Figure 9:**
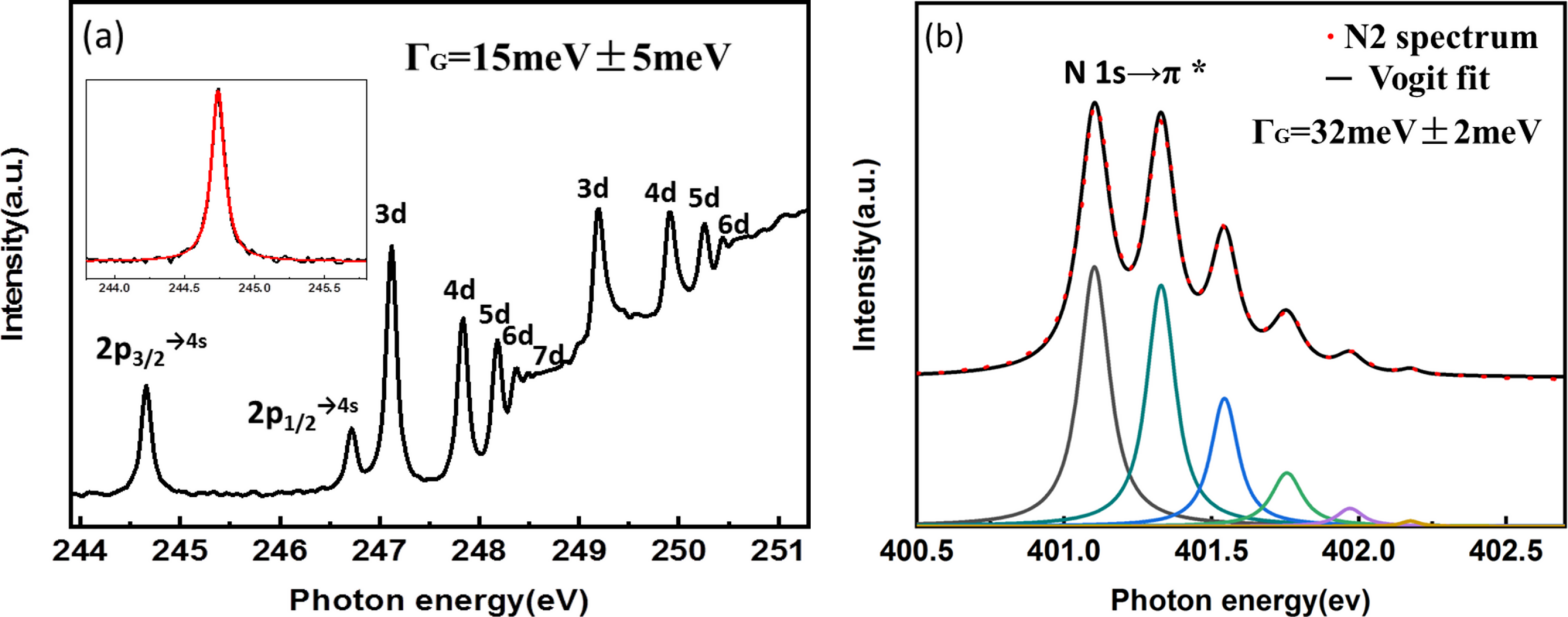
Ion yield spectra measured with the 1000 lines mm^−1^ grating.

Fig. 9 ▸(*a*) shows excitation spectra of argon measured with the 1000 lines mm^−1^ grating. The following Ar *L*_2,3_ absorption-edge transitions to the Rydberg levels were all observed: 2*p*_3/2_ → 4*s*, 3*d*, 4*d*, 5*d*, 6*d*, 7*d* and 2*p*_1/2_ → 4*s*, 3*d*, 4*d*, 5*d*, 6*d* (King *et al.*, 1977 ▸; Xue *et al.*, 2010 ▸; Meng *et al.*, 2019*b* ▸). The 2*p*_3/2_ → 4*s* transition (*h* = 244.39 eV) was used to characterize the energy resolution. When Voigt fitting in *Origin* using a Lorentzian linewidth ΔL = 111 meV (Prince *et al.*, 1999 ▸), the Gaussian broadening obtained is ΓG = 15 meV ± 5 meV, and the calculated resolving power of Ar is 1.6 × 10^4^.

Fig. 9 ▸(*b*) shows the excitation spectrum of nitro­gen measured with the 1000 lines mm^−1^ grating, in the range of the N 1*s*_1/2_ → π* excitation (Sodhi & Brion, 1984 ▸; Meng *et al.*, 2019*b* ▸). Six transition energies from N 1*s* (ν = 0) → π* (ν′: ν′ = 0–5) were clearly observed. The excitation spectrum was fitted using Lorentzian linewidth ΔL = 114 meV to obtain a Gaussian width ΓG = 31.5 meV ± 3 meV. We obtained a resolving power *R* = *E*/Δ*E* = 12730.

After the beamline upgrade, the energy resolving power has been significantly improved.

### 4.2. Flux at the sample

The photon flux tests were carried out at the sample location of the STXM endstation using an AXUV100 photodiode. The zone plate (ZP) and order-sorting aperture (OSA) in the STXM endstation were in the focusing mode during this test. The outer zone width and diameter of the ZP are 30 nm and 300 µm, respectively. The diameter of the OSA hole is 70 µm. During the flux test, the vacuum of the STXM chamber is greater than 1 × 10^−5^ Torr, and the size of the exit slits is 50 µm × 50 µm. The photocurrent (*I*_c_) of the 400 lines mm^−1^ grating was obtained at 244 eV for the Ar *L*_2,3_ spectra. The conversion formula between photocurrent and photon flux can be expressed as: flux = 3.66*I*_c_/(0.98*Ee*), where *e* and *E* represent the electronic charge and photon energy, respectively. The calculated flux is 2.45 × 10^9^ photons s^−1^ (*E*/Δ*E* = 6440 @ 244 eV). This photon flux has exceeded the theoretical calculated value of 5 × 10^8^ photons s^−1^, and has also exceeded the photon flux 1.8 × 10^8^ photons s^−1^ of the original beamline.

### 4.3. Energy range

**Figure 10:**
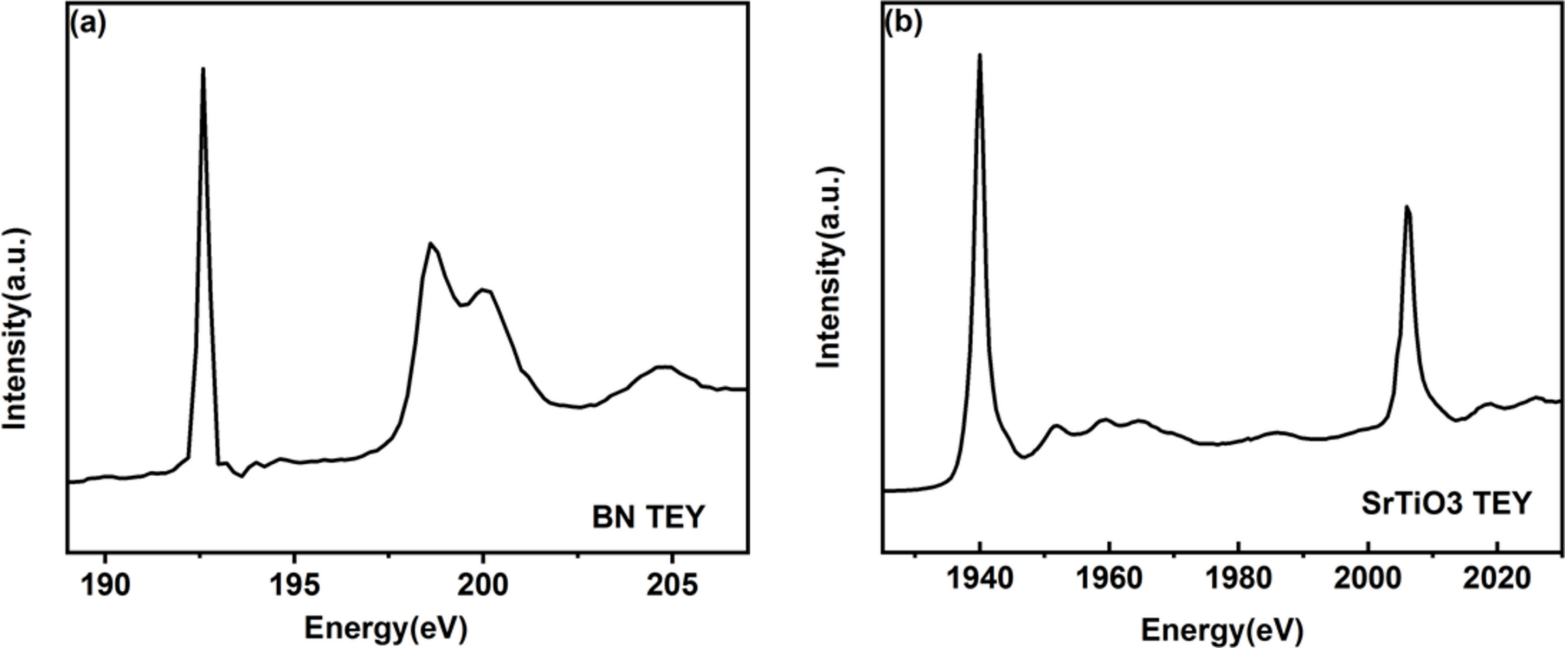
TEY of boron nitride (*a*) and strontium titanate (*b*).

The energy range of the BL08U1A beamline is designed to be between 200 eV and 2000 eV. The energy range is tested at the spectroscopic endstation. Boron nitride (BN) and strontium titanate (SrTiO_3_) are selected as low-energy and high-energy test samples. The TEY spectra of BN and SrTiO_3_ are shown in Fig. 10 ▸. The BN absorption peak is at 192.7 eV, verifying the low energy of the beamline. The SrTiO_3_ absorption peaks on the *L*_2_ and *L*_3_ sides are 1941 eV and 2008 eV, respectively, which verify the high energy of the beamline. Thus the energy range of this beamline is experimentally confirmed to be between 180 eV and 2000 eV.

### 4.4. Spatial resolution

**Figure 11:**
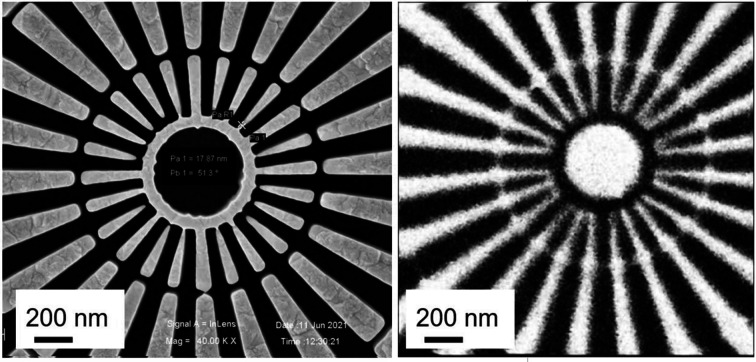
SEM (left) image and STXM (right) image of the magnified central part of a Siemens star.

Spatial resolution tests were carried out at the STXM endstation. Although the spatial resolution is mainly determined by the ZP, this test was performed after the beamline was upgraded. Fig. 6 ▸(*b*) shows the main components required for the STXM test: ZP, OSA, Siemens star and PMT detectors. The diameter and outer zone width of the ZP are 100 µm and 15 nm, respectively. The size of the OSA is 70 µm, and it can isolate the zero-order light but not the first-order light. A standard testing sample (an in-house-made Siemens star in Au with minimum feature size of 15 nm in the center) was selected as the test sample. The PMT was used as an experimental detector for STXM scanning imaging. Fig. 11 ▸ presents scanning electron microscope (SEM) and STXM images at 1200 eV with a spatial resolution better than 20 nm for the Siemens star (Tong *et al.*, 2023 ▸). This STXM image clearly shows the 15 nm wide lines in the center of the Siemens star.

### 4.5. Comparison with similar STXM beamlines

BL08U1A is a soft X-ray spectromicroscopy beamline that provides both linear (horizontal/vertical) and circular (right/left) polarization. The main experimental methods include STXM, STXM-ptychography and TEY spectroscopy. *In situ* magnetic field and low temperature experiments can also be performed. After the upgrade, BL08U1A has advantages in energy range, flux and energy resolving power over similar beamlines around the world. Table 2 ▸ summarizes details of three beamlines in terms of energy range, flux and energy resolving power. The data are sourced from the ALS, CLS and SLS websites.

## 5. Conclusion

The upgrade of SSRF BL08U1A restores and improves the beamline performance, providing high energy resolution and high photon flux for the experimental station. The upgrade included the following: the first mirror and the last mirror were changed from a cylindrical mirror (M1) and superannular mirror (M3) to elliptical cylinder mirrors (ECM1, ECM3); and the monochromator grating was upgraded from a plane-grating to a varied-line-space plane-grating. The photon flux in focused mode measured at the STXM endstation sample is 2.45 × 10^9^ photons s^−1^ (*E*/Δ*E* = 6440 @ 244 eV). The energy resolving powers were measured at 244 eV and 401 eV, with both being over 10000. The energy range was measured in the spectroscopic endstation, and can reach 180–2000 eV. This upgrade has achieved the goal of restoring the beamline performance.

## Figures and Tables

**Table 1:** Parameters of the beamline mirror

Mirror	Position (m)	Shape	Optical area (L × W) (mm)	Deflection/angle (°)	Coating	Slope error (tangential/sagittal) (µrad)	Roughness (nm)
ECM1	30	Elliptical cylindrical	230 × 25	1.72	Au	0.192/2	0.25
PM	32	Plane	430 × 60	Variable	Au	0.99/–	0.3
VLSG1	32	Plane	120 × 30	Variable	Au	0.172/–	0.37
VLSG2	32	Plane	120 × 30	Variable	Au	0.111/–	0.3
ECM3	34	Elliptical cylindrical	230 × 25	1.5	Au	0.32/2	0.21

**Table 2:** Comparison with other beamlines

Synchrotron	Country	Beamline	Energy range (eV)	Flux (photons s^−1^) at sample	Energy resolving power (*E*/Δ*E*)	Available equipment
SSRF	China	BL08U1A	180–2000	2.45 × 10^9^	16333 @ 244 eV	STXM
					12730 @ 401 eV	
ALS	US	5.3.2	250–2000	1 × 10^7^	≤5000	STXM
CLS	Canada	SM (10ID-1)	130–2500	10^8^	>10000	STXM
						X-PEEM
SLS	Switzerland	PolLux X07DA	250–1600	1 × 10^7^	>3000	STXM
